# Low Birth Weight Among Infants Born to Black Latina Women in the United States

**DOI:** 10.1007/s10995-018-2669-9

**Published:** 2019-01-02

**Authors:** Janardhan Mydam, Richard J. David, Kristin M. Rankin, James W. Collins

**Affiliations:** 1 Division of Neonatology, John H. Stroger, Jr. Hospital of Cook County, 1969 Ogden Avenue, Chicago, IL 60612 USA; 2 Department of Pediatrics, University of Illinois at Chicago, 1901 West Harrison Street, Chicago, IL 60612 USA; 3 Division of Epidemiology and Biostatistics, University of Illinois School of Public Health, 881 SPHPI MC 923 1603 W. Taylor Street, Chicago, IL 60612-4394 USA; 4 Department of Pediatrics, Feinberg School of Medicine, Northwestern University, 225 E Chicago Avenue, Box 45, Chicago, IL 60611 USA; 5 Division of Neonatology, Ann & Robert H. Lurie Children’s Hospital of Chicago, 225 E Chicago Avenue, Box 45, Chicago, IL 60611 USA

**Keywords:** Low birth weight, Disparities, Pregnancy risk factors, Race/ethnicity, Latina

## Abstract

*Objectives* To explore associations between race, nativity, and low birth weight (LBW) among Latina and non-Latina women, with special attention to the Black Latina subgroup. *Methods* Using US natality data from 2011 to 2013, we designed a population-based study to compare LBW (< 2500 g) rates across six groups of women with self-identified race (N = 7,865,264)—White and Black Latina, foreign-born and US-born; non-Latina Black; and non-Latina White—creating 4 models for analysis: unadjusted (Model 1) and adjusted for sociodemographic factors (Model 2), sociodemographic plus medical risk factors (Model 3), and Model 3 factors plus smoking (Model 4). *Results* Infant LBW rate for Black Latinas (7.9%) was higher than White Latinas (5.6%) and varied by nativity: US-born (8.9%) versus foreign-born (6.1%). Among all study groups, US-born Black Latinas’ LBW rate (8.9%) was second only to non-Latina Blacks (11.0%). In unadjusted Model 1, US-born Black Latinas had 81% (odds ratio [OR]: 1.81; 95% confidence interval [CI]: 1.74–1.88) and foreign-born Black Latinas had 22% (OR: 1.22; 95% CI 1.15–1.29) higher odds of LBW than non-Latina White women (reference group). However, in Model 2, ORs for foreign-born Black Latinas were nearly identical to non-Latina Whites (OR: 1.03; 95% CI 0.97–1.1), while US-born Black Latinas’ odds were still 47% higher (OR: 1.47; 95% CI 1.42–1.53). Model 3 ORs for each group were similar to Model 2. *Conclusions for Practice* A significant subgroup of Latina women self-identify as Black, and their LBW rates are higher than White Latinas. Black Latinas born in the United States fare worse than their foreign-born counterparts, implicating negative effects of Black race specific to the US context.

**Electronic supplementary material:**

The online version of this article (10.1007/s10995-018-2669-9) contains supplementary material, which is available to authorized users.

## Significance

*What is already known on this subject?* Considerable research has demonstrated heterogeneity in birth outcomes of Latina women in the United States when they are stratified by nativity, with better birth outcomes for foreign-born Latina women compared to their US-born counterparts. *What this study adds?* Few studies have explored the influence of race on birth outcomes of Latina women. Infants of Black Latina mothers are often not distinguished from infants born to other Latinas or non-Latina Blacks in clinical or epidemiological studies. With a growing number of Latinos in the United States, it is important to understand health differences among this population’s major subgroups.

Historically, research on birth outcomes of the Latino population in the United States has focused on maternal nativity, comparing immigrant (foreign-born) Latinas to Latinas born in the United States (US-born). Prior studies have consistently reported better birth outcomes for Latina immigrant women compared to US-born Latina women (Fuentes-Afflick et al. 1999; Collins and Shay 1994). However, findings vary when the Latina population is further subdivided by country of origin (Collins and Shay 1994; Acevedo-Garcia et al. 2007), level of acculturation (Scribner and Dwyer 1989), education level (Acevedo-Garcia et al. 2007), or length of US residence (Guendelman and English 1995). Such data suggests that similar heterogeneity of birth outcomes might also exist in low birth weight (LBW) rates among Latina women in the United States when subdivided by self-identified race.

We approach this study using the definition of race proposed by Jones (2001): “[R]ace is a social classification in our race-conscious society that conditions most aspects of our daily life experiences and results in profound differences in life chances.” Previous studies show a correlation between darker skin color and health, education, income, and employment disparities in Latino adults (Arce et al. 1987; Cuevas et al. 2016). The effects of these disparities on birth outcomes of racially diverse Latinas have been neglected. Moreover, research indicates that maternal experiences that predate conception influence birth outcomes (Collins et al. 2004; Dixon et al. 2012). The extent to which birth outcomes are influenced by a woman’s identity as a Black Latina, including the consequences of institutionalized, interpersonal, and internalized racism that may accompany that identity, has received very little attention.

Though self-reported race as well as the response to the “Mother of Hispanic Origin” field on the birth certificate are collected separately, the National Center for Health Statistics (NCHS) publications on birth outcomes do not report “Black Latina” as a separate racial-ethnic category (Ríos et al. 2014; CDC 2013; Martin et al. 2013). We identified very few studies of Black Latinas’ birth outcomes in the medical literature. In a 1993 study among Massachusetts Blacks by ethnic group, Black Latinas’ LBW rates were lower than non-Latina Blacks but significantly higher than non-Latina Whites (Friedman et al. 1993). Reichman and Kenney (1998), in their study of racially diverse Latinas in New Jersey, and, more recently, Bediako et al. (2015), in a larger, population-based study, found higher LBW prevalences for Black Latinas compared to White Latinas, with highest prevalence for non-Latina Blacks (Reichman and Kenney 1998; Bediako et al. 2015). Two additional studies addressed birth outcomes in Black Latinas but appeared in social science journals. Henry-Sanchez and Geronimus (2013) found significant differences in infant mortality rates between White and Black Latinas in pre- and post-surfactant periods, and Green (2014) found Black Latino newborns weighed more, on average, than non-Latino Blacks, without specifying LBW. Of these studies, only Green reported separate results for US-born versus foreign-born Latinas. The extent to which nativity, sociodemographic factors, and reproductive risk factors affect the relationship between maternal race and infant LBW rates of Latina women needs more investigation.

Therefore, we designed a population-based study to test the hypothesis that LBW rates of infants born to Latina women vary when women are stratified by race. The primary objective of the present study is to explore associations between race, nativity, and LBW among Latina women, adjusting for sociodemographic and medical risk factors. As a secondary objective, we compare LBW rates of Latina Black and White women with those of non-Latina Black and White women.

## Methods

### Study Data

**Fig. 1:**
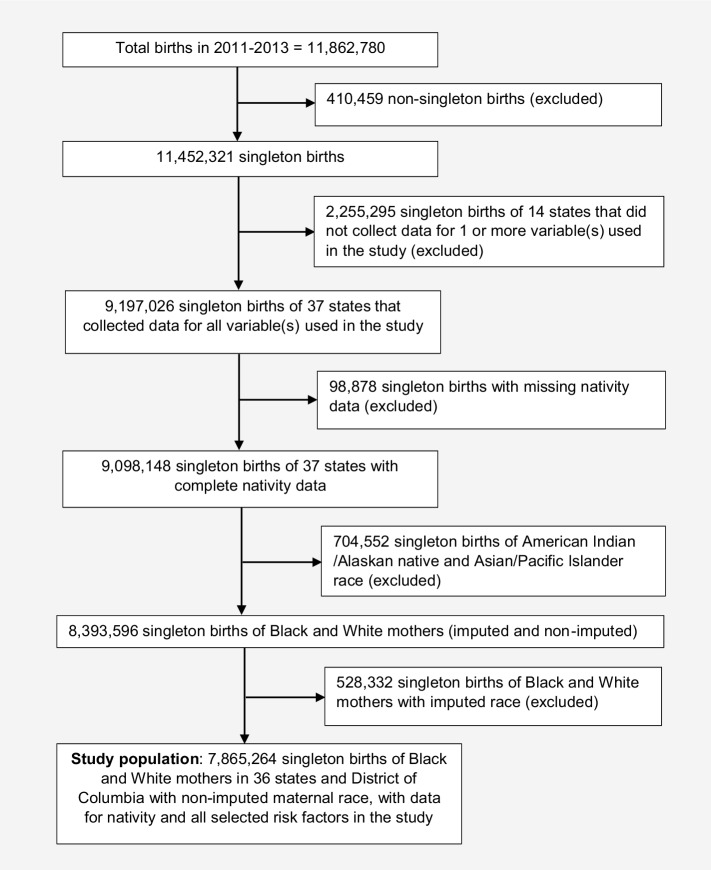
Flow chart of study population selection indicating steps of inclusion and exclusion of data from the 2011–2013 National Centers for Health Statistics (NCHS) natality database. States excluded due to missing data: AL, AR, AK, AZ, CT, HI, ME, NJ, RI, WV, MI, MS, GA and VA. States included, with complete data: CA, CO, DE, FL, IA, ID, IL, IN, KS, KY, LA, MA, MD, MN, MO, MT, NC, ND, NE, NH, NM, NV, NY, OH, OK, OR, PA, SC, SD, TN, TX, UT, VT, WA, WI, WY, and the District of Columbia

Upon special request and after complying with CDC data use agreements, we accessed National Centers for Health Statistics (NCHS) natality data for 11,862,780 live births from the Centers for Disease Control and Prevention (CDC) for infants born in the United States from 2011 to 2013. Elements extracted from the NCHS data included, but were not limited to, birth outcome, sex of infant, paternal acknowledgement, and the following maternal characteristics: self-reported race, nativity, ethnicity, age, marital status, education, parity, medical disease during pregnancy, cigarette smoking, pre-pregnancy BMI, timing of prenatal care initiation, and Special Supplemental Nutrition for Women, Infants and Children (WIC) receipt. We excluded all non-singleton births; singleton live births from 14 states that either did not collect data for one or more study variables or collected data in a nonstandard format (MI [Osterman et al. 2013] and GA, for smoking; Online Resource Table 1); infants with missing nativity data; women identified as Asian/Pacific Islander and Indian/Alaskan Native race; and infants born to Black and White women whose race had been assigned by imputation (Fig. 1). The NCHS natality data “flags” the race field to indicate when a woman’s race has been imputed (CDC 2013).

We excluded women whose race was assigned by imputation because our goal was to investigate birth outcomes of clearly self-identified Black Latinas; although Jones (2001) argues convincingly that a person’s socially defined racial category is determined by others, not by herself, we consider self-identified race the best indicator of this variable available in vital records.

The following are the percentage of women in each race-nativity group whose data was excluded because of imputed race: 40.5% (22,918) of US-born Black Latinas; 63.4% (32,313) of foreign-born Black Latinas; 15.2% (157,683) of US-born White Latinas; 26.1% (294,533) of foreign-born White Latinas; 0.3% (15,806) of non-Latina Whites; and 0.4% (5079) of non-Latina Blacks (Online Resource Table 2).

Race classification of Latino populations in US census and NCHS natality data (Humes et al. 2011; Ríos et al. 2014; CDC 2013), including the imputation process (CDC 2013), is explained in the Online Resource Appendix.

In the analyzed dataset of 7,865,264 births, only 5,829 (0.1%) birth records were missing birth weight (Online Resource Table 3). Among analyzed risk factors (independent variables), maternal age, marital status, and sex of infant had no missing values; BMI had the highest percentage of missing values (3.2%) (Online Resource Table 3).

### Variables, Definitions, and Group Classification

The primary outcome variable LBW was defined as a birth weight less than 2500 g regardless of gestational age. Self-identified race was defined as Black or White as recorded on the infant’s birth certificate (CDC 2013). Maternal nativity variables included foreign-born and US-born; US-born included women born within the 50 states including New York City and the District of Columbia (CDC 2013). Our independent variable of interest, or “Group” variable, consists of 6 study groups representing categories of maternal race, ethnicity, and nativity: US- and foreign-born Black Latinas, US- and foreign-born White Latinas, non-Latina Blacks, and non-Latina Whites. For comparability with most other studies, infants of non-Latina Black and White women were not further classified by nativity. Our other independent variables included sociodemographic (marital status, paternal acknowledgement [social]; WIC receipt, a proxy for low income, and maternal education [socioeconomic; Shavers 2007]; maternal age [demographic]), and medical (sex of infant, medical disease during pregnancy, parity, prenatal care initiation, smoking, pre-pregnancy BMI) factors.

Maternal characteristics were categorized as follows: age, < 20 and ≥ 20 years; education, < 12 and ≥ 12 years; pre-pregnancy BMI, underweight (< 18.5), normal (18.5–24.9), overweight (25.0–29.0), or obese (> 30.0; CDC 2013); parity, primiparous, low (1–3), or high (> 3). Prenatal care initiation was based on trimester of entry. Medical disease during pregnancy included gestational diabetes, pre-pregnancy diabetes, gestational hypertension, pre-pregnancy hypertension, and eclampsia.

### Statistical Analysis

Using SAS version 9.4 software (SAS Institute, Inc., Cary, North Carolina) for statistical analysis, we conducted Chi square (*χ*^2^) tests to explore statistical differences in maternal characteristics by maternal race, ethnicity, and nativity, and used Cramer’s V statistic as a measure of effect size to assess the strength of observed differences. To estimate the association between the Group variable (race, ethnicity, and nativity) and LBW, we created 4 regression models; non-Latina White women served as the reference group. Model 1, a crude analysis (unadjusted model) included only the Group variable; ORs from Model 1 were used as a measure of effect size for the strength of the association between LBW and the Group variable. Model 2 adjusted for confounding effects of measured sociodemographic factors (maternal age, education, marital status, paternal acknowledgment, and WIC receipt) with further analysis to assess potential mediators using Baron and Kenny (1986) method. None of the variables demonstrated complete mediator effect; however, maternal education, marital status, and paternal acknowledgement showed partial mediator effects on the Group variable (Online Resource Table 4). Model 3 added to Model 2 the major medical risk factors for LBW other than smoking (prenatal care initiation, pre-pregnancy BMI, parity, infant’s sex, and medical disease during pregnancy). Logistic regression analyses showed a strong impact of smoking on LBW, specifically addressed in Model 4, which included all Model 3 factors plus smoking.

The entire analysis was repeated after re-incorporating births with imputed race (n = 8,393,596) to assess the sensitivity of our findings to our exclusion of observations with imputed race. We also compared the distribution of risk factors among Black Latina women with imputed versus non-imputed race, stratified by nativity, to assess potential bias if excluded data was not random.

## Results

Among all singleton births to women self-identifying as either Black or White race, 1,768,318 (22.5%) identified as Latina. Of births to self-identified Latinas, 52,361 (3.0%) women identified as Black and 1,715,957 (97%) identified as White. Among infants of Latina Black and White women, about half (51.7%) had mothers born in the United States. In contrast, 84.5% of infants born to non-Latina Black and 94% of those born to non-Latina White women had US-born mothers (Online Resource Table 1).

While LBW rates of US-born and foreign-born non-Latina Whites were not equal (5.2% and 4.2%, respectively), they were combined for analysis for simplicity and comparability with other studies (Fuentes-Afflick et al. 1999; Acevedo-Garcia 2007) regardless of nativity (Online Resource Table 5). Non-Latina Blacks had starker differences between LBW rates by nativity (11.6% and 7.6% for US-born and foreign-born women respectively); because this group was not our focus and US-born women dominated, we combined them for simplicity (Online Resource Table 5).

Table 1 shows the prevalence of risk factors for infants of Black and White Latina women by nativity, compared to those of non-Latina Black and White women (Table 1). While non-Latina Black women had the highest overall risk profile, Black Latinas had the second highest prevalence for a number of risk factors. US-born Black Latinas had the highest percent of teen births and unmarried status and the second-highest percent of unmarried without paternal acknowledgement status. Foreign-born Black Latinas had the second highest percent of low maternal education, WIC receipt, and medical disease during pregnancy. White Latinas, by contrast, generally had a lower risk profile, except for low educational attainment among the foreign-born and the highest percent of WIC receipt. Of note, all Latina groups had lower smoking percentages than non-Latina women. White non-Latinas had the highest percent of smoking (13%). All risk factors differed significantly across the six groups (P < 0.001). The effect size was greatest for maternal education, marital status, unmarried without paternal acknowledgement, and WIC receipt (medium effect size; Cramer’s V = 0.3–0.4; Table 1).

**Table 1:** Distribution (%) of selected risk factors among Black Latina US-born, Black Latina foreign-born, White Latina US-born, White Latina foreign-born, non-Latina White, and non-Latina Black mothers, United States, 2011–2013

Risk factor	Black Latina		White Latina		Non-Latina White *n* = 4,885,806 %	Non-Latina Black *n* = 1,211,140 %	Effect size^c^ (lower dimension^d^)
	US born *n* = 33,690 %	Foreign born *n* = 18,671 %	US born *n* = 880,071 %	Foreign born *n* = 835,886 %			
Maternal age < 20 years	26^a,b^	10	23	9	9	19	0.16 (2)^f^
High parity	12	11	12	19	10	15	0.09 (3)^f^
Maternal education < 12 years	22	33	23	48	9	20	0.32 (2)^g^
Unmarried	75	61	57	47	30	72	0.33 (2)^g^
Unmarried and no paternal acknowledgement	25	16	14	9	8	32	0.26 (3)^g^
No WIC recipient	32	27	35	25	68	33	0.36 (2)^g^
Medical disease during pregnancy	9	11	9	10	11	13	0.04 (2)^e^
No first trimester initiation of prenatal care	34	31	28	31	21	37	0.13 (2)^f^
Smoke cigarettes	8	1	3	0.4	13	8	0.16 (2)^f^
Normal BMI (18.5–24.9)	40	46	41	44	50	37	0.07 (4)^f^

*WIC* special supplemental nutrition for women, infants and children

Findings from our analysis re-incorporating births with imputed race are shown in a supplementary table (Online Resource Table 8)

^a^The *P-*value of *χ*^2^ test of association among six groups of women is < 0.0001 for all risk factors

^b^Percentages for all risk factors are rounded to the nearest integer

^c^Cramer’s V statistic is used as a measure of effect size

^d^Lower dimension is the minimum(r,c) for a (r × c) cross table

^e^Negligible effect size

^f^Small effect size

^g^Medium effect size

**Fig. 2:**
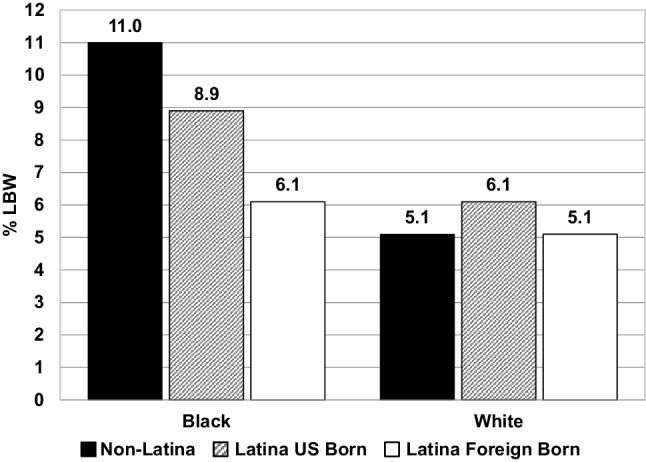
Distribution of infant low birth weight rates among Black and White mothers according to nativity and Latina/non-Latina ethnicity, United States, 2011–2013. Findings from our analysis re-incorporating births with imputed race are shown in a supplementary figure (Online Resource Fig. 1)

Focusing on Latinas’ nativity, the LBW rate for US-born women was 6.2% versus 5.1% for foreign-born women (Online Resource Table 6). Turning attention to race, the LBW rate for Black Latina women was 7.9% versus 5.6% for White Latina women (Online Resource Table 7). When Latina women were stratified by both nativity and race, US-born Black Latinas had the highest LBW rates (8.9%) and foreign-born White Latinas had the lowest (5.1%). Non-Latina Black and White women had LBW rates of 11.0% and 5.1% respectively (Fig. 2).

Table 2 shows results of the logistic regression analysis. From Model 1, the unadjusted model for the association between infant LBW and the Group variable (with corresponding ORs as measures of effect size) to Model 4, adjusted for all selected risk factors, non-Latina Black women, US-born Black Latinas, and US-born White Latinas all had higher LBW odds compared to non-Latina White women (*P* < 0.001). For each group, the ORs were attenuated slightly once adjusted for maternal sociodemographic covariates (Model 2) and additionally for medical risk factors except smoking (Model 3). The ORs increased again when we added smoking to the fully adjusted model (Model 4).

**Table 2:** Odds ratios for the effect of maternal ethnicity, nativity, and race on infant low birth weight among Latina and non-Latina Black and White mothers, adjusting for different sets of risk factors in multivariable logistic regression models, United States, 2011–2013

Study groups by race, ethnicity, and nativity	Model 1^a^ *n* = 7,859,435		Model 2^b^ *n* = 7,677,906		Model 3^c^ *n* = 7,274,641		Model 4^d^ *n* = 7,252,226	
	OR	95% CI	OR	95% CI	OR	95% CI	OR	95% CI
Non-Latina White^e^	1.00		1.00		1.00		1.00	
Non-Latina Black	2.31***	2.29, 2.32	1.88***	1.86, 1.89	1.93***	1.91, 1.94	2.21***	2.19, 2.23
Black Latina US-born	1.81***	1.74, 1.88	1.47***	1.42, 1.53	1.56***	1.50, 1.62	1.78***	1.71, 1.85
Black Latina foreign-born	1.22***	1.15, 1.29	1.03	0.97, 1.10	1.07*	1.00, 1.14	1.30***	1.22, 1.39
White Latina US-born	1.21***	1.20, 1.23	1.06***	1.05, 1.07	1.15***	1.14, 1.16	1.34***	1.33, 1.35
White Latina foreign-born	1.00	0.99, 1.02	0.87***	0.86, 0.88	0.92***	0.91, 0.93	1.13***	1.11, 1.14

*CI* confidence interval, *OR* odds ratio, *WIC* special supplemental nutrition for women, infants and children

Findings from our analysis re-incorporating births with imputed race are shown in a supplementary table (Online Resource Table 9)

*P < 0.05; **P < 0.01; ***P < 0.001

^a^Model 1, a crude analysis (unadjusted model), included only the Group variable (the Group variable represents the six study groups of race, ethnicity, and nativity categories)

^b^Model 2 adjusted for maternal age, maternal education, marital status, paternal acknowledgment, and WIC recipient

^c^Model 3 adjusted for Model 2 covariates plus sex of infant, first trimester initiation, parity, mother’s medical disease during pregnancy, and body mass index (BMI)

^d^Model 4 adjusted for Model 3 covariates plus cigarette smoking

^e^Reference group

Even after adjustment, US-born Black Latinas had greater than 50% higher odds of LBW compared to non-Latina Whites, and odds for non-Latina Blacks were approximately twofold higher. While foreign-born Black Latinas had 22% higher LBW odds compared to non-Latina Whites (OR: 1.22; 95% CI 1.15, 1.29) in the unadjusted model (Model 1), adjustment for sociodemographic and then also medical risk factors resulted in ORs close to the null (OR: 1.03; 95% CI 0.97, 1.1 and OR: 1.07; 95% CI 1.0, 1.14 for Models 2 and 3, respectively). In adjusted Models 2 and 3, foreign-born White Latinas had significantly lower odds of LBW compared to non-Latina Whites, with ORs of 0.87 (95% CI 0.86, 0.88) for Model 2 and 0.92 (95% CI 0.91, 0.93) for Model 3. The added adjustment for smoking status in Model 4 resulted in ORs for LBW increasing for all groups, given that the referent group—non-Latina White women—was more likely to smoke (Table 2).

Findings from our sensitivity analysis re-incorporating births with imputed race were not meaningfully different than those reported above (Online Resource Tables 8–9 and Online Resource Fig. 1).

## Discussion

In this population-based study, we add novel information about the birth outcomes of Latina women by reporting differences in Latinas’ LBW rates by both nativity and race. In our analysis, US-born Black Latinas had the highest LBW rates among all Latina groups, and the second-highest LBW rates in the sample, second only to non-Latina Black women. In contrast, foreign-born Black Latinas had similar odds of LBW as non-Latina Whites after we adjusted for sociodemographic covariates (Model 2), and only borderline higher odds after also adjusting for medical risk factors (excluding smoking; Model 3). These two findings contradict studies that attribute poor birth outcomes, in part, to Black race itself (Straughen et al. 2015; Swamy et al. 2011), implying a biologic or genetic basis for racial health inequities. Our findings, like previous work (Jones 2001; Castrillio et al. 2014) indicate that medical research presuming that skin color is linked to genetic differences of medical importance between socially defined “races” is misguided.

The observed differences in LBW rates for White and Black Latina women are consistent with reports by Henry-Sanchez and Geronimus (2013), who found that LBW rates of White Latina and Black Latina women were 5.2% and 8.7% (respectively) in 1989–1990, and 5.3% and 8.4% (respectively) in 1995–1999, and Bediako et al. (2015), who found that, after adjusting for sociodemographic and medical risk factors, Black Latinas were more likely than non-Black Latinas and less likely than non-Latina Blacks to give birth to LBW infants. Bediako did not consider maternal nativity as a covariate; although Henry-Sanchez et al. included maternal nativity as a covariate, they did not report separate results for US- and foreign-born women. Similarly, the study by Friedman et al. (1993), with only 575 Black Latinas, was not able to stratify results for Black Latinas by nativity. Green (2014), using national data, found higher mean birth weights for Black Latinas compared to non-Latina Blacks and, like our study, found that foreign-born Black Latinas had heavier babies than their US-born counterparts. However, she did not evaluate LBW rates, which are more closely tied to infant mortality, or compare Black Latinas with White women, Latina or non-Latina.

In our study, LBW rates of both foreign-born and US-born Black Latinas were lower than those of non-Latina Blacks, consistent with Green (above) and Reichman and Kenney (1998), who found that Black Latinas (n = 2127) had higher LBW rates than White Latinas, but still much lower than non-Latina Blacks. The better birth outcomes of Black Latinas compared to non-Latina Blacks may be attributable, in part, to favorable social, cultural, or lifestyle elements within Latina communities (Scribner and Dwyer 1989; Mason et al. 2011). However, when US- and foreign-born Black Latinas are compared separately to non-Latina Black women, the gap narrows for US-born Black Latinas and widens for foreign-born Black Latinas, indicating a significant loss of the protective effect of Latino ethnicity after one or more generations in the United States.

The higher LBW rates we observed for Black Latinas, regardless of nativity, when compared to their White Latina counterparts, demonstrates a significant association of Black race and LBW among the Latina population. Racism—institutional, cultural, and interpersonal—negatively affects health outcomes (Williams and Mohammed 2013; Jones 2001), including birth outcomes (Williams and Mohammed 2013). The persistence of higher LBW rates among Black Latinas in our study after adjusting for socioeconomic factors (eg, WIC receipt, education) corroborates observations that racial disparities in health persist across all socioeconomic levels (Williams et al. 2010) and highlights the adverse health effects of cultural and interpersonal racism. Potential pathways include effects of provider discrimination on prenatal care quality (Williams and Mohammed 2013; Jones 2001); responses to cultural racism such as stereotype threat and internalized racism (Williams and Mohammed 2013) that manifest as health care avoidance or poor adherence (Aronson et al. 2013); and the physiologic effects of chronic stress from perceived racism and discrimination on fetal growth and birth weight (Dunkel-Schetter 2011). Lifetime experience of racial discrimination has been reported as an independent risk factor for LBW (Dixon et al. 2012) and preterm delivery (Collins et al. 2004). Finally, mental health issues affecting darker-skinned Latinas (Montalvo 2005) may add to the complex mechanisms underlying the racial disparity reflected in LBW rates of Black and White Latinas in our study. Colorism—within-group discrimination based on skin tone, with the understanding that lighter is better (Montalvo 2005)—runs deep in Latino culture (Chavez-Dueñas et al. 2014). Disparities in education, income, employment, and health in Latin American countries based on a hierarchy of skin color are well-documented (Telles 2014; Haywood 2017), with Afro-Latinos at the bottom of the pyramid (Chavez-Dueñas et al. 2014).

However, when we compared all 6 groups, including Latina and non-Latina women, across race and nativity, the odds of LBW among foreign-born Black Latinas are better (Model 3) and no different (Models 1, 2, and 4) than LBW odds for US-born White Latinas. Perhaps more significantly, LBW odds for foreign-born Black Latinas were no different (Models 2 and 3) than odds for non-Latina Whites. Our findings strongly suggest that the negative effect of race on birth outcomes in Black Latina women is dependent on the US context for full expression. Because skin-tone discrimination persists within immigrant Latino communities (Haywood 2017), poor birth outcomes among US-born Black Latinas may reflect health effects of discrimination both inside and outside the Latino community. Further multidisciplinary investigations are needed to unravel the specific factors responsible for these observations.

The observed increase in LBW odds for Latinas when smoking during pregnancy was added in Model 4 is not surprising considering the extremely wide gap in exposure to this risk factor between Latinas and non-Latina White women and suggests the LBW disparities between the other subgroups and non-Latina White women would be even wider if smoking rates were the same across the groups.

The diversity observed in LBW rates across subgroups of Latina women strongly suggests that future studies evaluating birth outcomes of Latina women should consider self-identified race as well as nativity.

### Limitations

Although our population-based study uses a large dataset of US births from 2011 to 2013 and complete Latina ethnicity by race, there are some limitations. First, we focused our analysis on women with clearly self-identified race, excluding approximately 50% of presumed Black Latinas (Online Resource Table 2). To explore potential bias of excluded data we compared the distribution of covariates from Models 2–4 of imputed versus non-imputed Black Latinas. All but three covariates were randomly distributed (Online Resource Table 10). Because our analysis of combined (imputed and non-imputed) data yielded very similar results as our analysis limited to women with clearly self-identified race (Online Resource Tables 8–9, 11–12 and Online Resource Fig. 1) a bias effect created by covariate imbalance is unlikely.

Second, women from Mexico, Puerto Rico, Cuba, Central America, South America, and other Latino countries of origin are not racially, socially, or politically homogeneous. Categorizing them as a single ethnic group could mask differences that affect maternal health and, consequently, birth outcomes. For example, LBW rates for foreign-born Black Latinas might vary depending on the intensity of color tone discrimination in their home country.

Third, we were unable to assess other lifelong experiences such as length of maternal residence in the United States (ie, acculturation), the women’s intrauterine environment, or class in home country, a theoretical contributor to foreign-born Black Latinas’ advantage over their US-born counterparts. Assessing class is a challenge; assessment variables such as multileveled occupation and education measures (Krieger et al. 1997) were not available in our natality data. Studies of Black Latinas’ birth outcomes designed to assess class are warranted.

Finally, LBW may be a consequence of early delivery and/or poor fetal growth, whose etiologies are not identical. Therefore, future investigations to examine these outcomes separately would enhance our understanding of potential pathways (Dunkel-Schetter 2011).

In summary, Latina women identify themselves as different races in US health data, and experiences associated with those racial identities, such as institutionalized, interpersonal, and internalized racism, may be responsible for the observed disparities in LBW, especially after a generation in this country. The mechanisms underlying these findings are probably complex and myriad. We encourage researchers to develop conceptual models to elucidate the effect of race on Latina women’s birth outcomes over time.

## Electronic supplementary material

Below is the link to the electronic supplementary material.

Supplementary material 1 (DOCX 330 KB)
