# Supplementary material for: Low Birth Weight Among Infants Born to Black Latina Women in the United States
Source: Matern Child Health J. 2019 Jan 2;23(4):538–46. doi: 10.1007/s10995-018-2669-9 (PMC6439288; doi:10.1007/s10995-018-2669-9)
Supplement: Supplementary file 1 — Supplementary material 1 (DOCX 330 KB) [file 10995_2018_2669_MOESM1_ESM.docx]

**Electronic Supplementary Material**

**Low Birth Weight Among Infants Born to Black Latina Women in the United States**

**Maternal and Child Health Journal**

**Authors: Janardhan Mydam**, **Richard J. David, Kristin M. Rankin, James W. Collins**

Janardhan Mydam (corresponding author)

Division of Neonatology, John H. Stroger, Jr. Hospital of Cook County

1969 Ogden Avenue, Chicago, IL, 60612

mydamj@gmail.com

jmydam@cookcountyhhs.org

313-969-9720

Richard J. David

Department of Pediatrics, University of Illinois at Chicago, Chicago, Illinois

Division of Neonatology, John H. Stroger, Jr. Hospital of Cook County

1901 West Harrison Street, Chicago, IL, 60612

rdavid@uic.edu

Kristin M. Rankin

Division of Epidemiology and Biostatistics, University of Illinois School of Public Health, Chicago, Illinois

881 SPHPI MC 923 1603 W. Taylor Street
Chicago, IL, 60612-4394

krankin@uic.edu

James W. Collins

Department of Pediatrics, Feinberg School of Medicine, Northwestern University, and Division of Neonatology, Ann & Robert H. Lurie Children’s Hospital of Chicago, Chicago, Illinois

Ann & Robert H. Lurie Children's Hospital of Chicago Box 45
225 E Chicago Avenue
Chicago IL 60611

[jcollins@northwestern.edu](mailto:jcollins@northwestern.edu)

**Corresponding author’s e-mail:**

mydamj@gmail.com

jmydam@cookcountyhhs.org

**Online Resource** (**Supplementary) Tables 1-12 and Online Figure 1**

**Online Resource Table 1.** Distribution of Total Singleton Births According to Mother’s Nativity, Ethnicity, and Race (Non-Imputed) in Restricted and Unrestricted NCHS Natality Data, United States, 2011-2013

| **Mother's Nativity** | **Ethnicity** | **Race** | **Unrestricted Data^a^**  ***n* (%)** | **Restricted**  **Data^b^**  ***n* (%)** |
| --- | --- | --- | --- | --- |
| US Born | Latina | Black | 41,936 (0.4) | 33,690 (0.4) |
|  |  | White | 996,100 (9.3) | 880,071 (10.3) |
|  |  | Asian/Pacific Islander | 11,947 (0.1) | 6,783 (0.1) |
|  |  | Indian/Alaskan Native | 10,933 (0.1) | 9,688 (0.1) |
|  |  | **Sub-Total** | **1060,916 (9.9)** | **930,232** (**10.9**) |
|  | Non-Latina | Black | 1,431,447 (13.4) | 1,023,561 (12.0) |
|  |  | White | 5,759,737 (53.8) | 4,592,956 (53.7) |
|  |  | Asian/Pacific Islander | 146,607 (1.4) | 111,734 (1.3) |
|  |  | Indian/Alaskan Native | 111,445 (1.0) | 81,490 (1.0) |
|  |  | **Subtotal** | **7,449,236 (69.6)** | **5,809,741** (**68.0**) |
|  | **Total US Born** |  | **8,510,152 (79.5)** | **6,739,973** (**78.8**) |
| Foreign Born | Latina | Black | 25,653 (0.2) | 18,671 (0.2) |
|  |  | White | 985,130 (9.2) | 835,886 (9.8) |
|  |  | Asian/Pacific Islander | 4,556 (0.0) | 3,357 (0.0) |
|  |  | Indian/Alaskan Native | 2,073 (0.0) | 1,847 (0.0) |
|  |  | **Subtotal** | **1,017,412 (9.5)** | **859,761** (**10.1**) |
|  | Non-Latina | Black | 230,200 (2.2) | 187,579 (2.2) |
|  |  | White | 367,504 (3.4) | 292,850 (3.4) |
|  |  | Asian/Pacific Islander | 573,428 (5.4) | 468,972 (5.5) |
|  |  | Indian/Alaskan Native | 1,823 (0.0) | 1,028 (0.0) |
|  |  | **Sub total** | **1,172,955 (11.0)** | **950,429** (**11.1**) |
|  | **Total Foreign Born** |  | **2,190,367 (20.5)** | **1,810,190** (**21.2**) |
| **Grand total** |  |  | **10,700,519 (100)** | **8,550,163** (**100**) |

^a^Unrestricted data contains 2011-2013 NCHS natality data for singleton live births of US-born and foreign-born mothers (excluding unknown/missing nativity) for all 50 states (including the 14 states with inadequate data) plus the District of Columbia.

^b^Restricted data contains 2011-2013 NCHS natality data for singleton live births of US-born and foreign-born mothers (excluding unknown/missing nativity) for 36 states plus District of Columbia; the 14 states excluded from the study either did not collect data for one or more study variable(s) or collected data in a nonstandard format (MI [Osterman et al. 2013] and GA, for smoking).

**Online Resource Table 2.** Distribution of Non-Imputed and Imputed Race Among Latina and Non-Latina Mothers and Latina Mothers Subdivided by Race and Nativity, in Unrestricted and Restricted NCHS Singleton Natality Data, United States, 2011-2013

| **Group** | **Unrestricted Data^a^**  **Maternal Race** | |  | **Restricted Data^b^**  **Maternal Race** | |
| --- | --- | --- | --- | --- | --- |
|  | Non-Imputed  *n* (%) | Imputed  *n* (%) |  | Non-Imputed  *n* (%) | Imputed  *n* (%) |
| Latina | 2,078,328 (77.7) | 597,388 (22.3) |  | 1,789,993 (77.3) | 524,658 (22.7) |
| Black Latina | 67,589 (51.0) | 64,901 (49.0) |  | 52,361 (48.7) | 55,231 (51.3) |
| White Latina | 1,981,230 (79.5) | 512,388 (20.6) |  | 1,715,957 (79.1) | 452,216 (20.9) |
| Non-Latina White | 6,127,241 (99.6) | 24,775 (0.4) |  | 4,885,806 (99.7) | 15,806 (0.3) |
| Non-Latina Black | 1,661,647 (99.5) | 8,309 (0.5) |  | 1,211,140 (99.6) | 5,079 (0.4) |
| Black Latina US-Born | 41,936 (61.6) | 26,133 (38.4) |  | 33,690 (59.5) | 22,918 (40.5) |
| Black Latina Foreign-Born | 25,653 (39.8) | 38,768 (60.2) |  | 18,671 (36.6) | 32,313 (63.4) |
| White Latina US-Born | 996,100 (85.3) | 171,870 (14.7) |  | 880,071 (84.8) | 157,683 (15.2) |
| White Latina Foreign-Born | 985,130 (74.3) | 340,518 (25.7) |  | 835,886 (73.9) | 294,533 (26.1) |

Abbreviations: NCHS, National Center for Health Statistics

^a^Unrestricted data contains 2011-2013 NCHS natality data for singleton live births of US-born and foreign-born mothers (excluding unknown/missing nativity) for all 50 states plus District of Columbia (including the 14 states with inadequate data).

^b^Restricted data contains 2011-2013 NCHS natality data for singleton live births of US-born and foreign-born mothers (excluding unknown/missing nativity) for 36 states plus District of Columbia; the 14 states excluded from the study either did not collect data for one or more study variable(s) or collected data in a nonstandard format (MI [Osterman et al. 2013] and GA, for smoking).

**Online Resource Table 3.** Summary of Analyzed and Missing Observations of Selected Variables in the Study Population,^a^ United States, 2011-2013

| **Variable** | **Observations** | |
| --- | --- | --- |
|  | **Analyzed^b^**  ***n* (%)** | **Missing**  ***n* (%)** |
| Low birth weight | 7,859,435 (99.9) | 5,829 (0.1) |
| Race and nativity | 7,865,264 (100.0) | 0 (0.0) |
| Maternal age | 7,865,264 (100.0) | 0 (0.0) |
| Maternal education | 7,818,503 (99.4) | 46,761 (0.6) |
| Marital status | 7,865,264 (100.0) | 0 (0.0) |
| Parity | 7,829,463 (99.5) | 35,801 (0.5) |
| First trimester initiation of prenatal care | 7,637,237 (97.1) | 228,027 (2.9) |
| Paternal acknowledgement | 7,843,087 (99.7) | 22,177 (0.3) |
| WIC recipient | 7,739,355 (98.4) | 125,909 (1.6) |
| Gender of infant | 7,865,264 (100.0) | 0 (0.0) |
| Medical disease during pregnancy | 7,832,247 (99.6) | 33,017 (0.4) |
| Body mass index | 7,612,175 (96.8) | 253,089 (3.2) |
| Smoking cigarettes | 7,809,950 (99.3) | 55,314 (0.7) |

Abbreviation: WIC, Special Supplemental Nutrition for Women, Infants and Children

^a^Study population: 7,865,264 singleton births of Black and White women in 36 states and District of Columbia with non-imputed maternal race, with data for nativity and all selected risk factors in the study (Main manuscript Figure 1).

_._^b^No missing observations.

**Online Resource Table 4.** Mediation Analysis to Identify Mediator Variables (Among Sociodemographic Risk Factors Adjusted in Model 2) That Mediate the Relationship Between LBW (*Y*) and Race-Ethnicity-Nativity (*X*) to predict LBW in the Study Population,^a^ United States, 2011-2013

| Case | Independent (*X*)/ Mediator (*M*) | Traditional Mediation Steps | | | |
| --- | --- | --- | --- | --- | --- |
|  |  | (1) Logit(*Y*)=b0+b_1_*X+e*  b^*^ (*P*-value) | (2) Logit(*M*)=b0+b_1_*X+e*  b (*P*-value) | (3) Logit(*Y*)=b0+b_1_*M+e*  b (*P*-value) | (4) Logit(*Y*)=b0+b_1_X+b_2_*M+e*  b (*P*-value) |
| 1 | Independent (*X*): Race- Ethnicity-Nativity (Ref: NLW) |  |  |  |  |
|  | BLFB | 0.1974 (<.0001) | 0.0595 (0.0173) |  | 0.1961 (<.0001) |
|  | BLUSB | 0.5937 (<.0001) | -1.2629 (<.0001) |  | 0.5504 (<.0001) |
|  | WLFB | .00392 (0.4663) | -0.0301 (<.0001) |  | 0.00326 (0.5441) |
|  | WLUSB | 0.1935 (<.0001) | -1.1282 (<.0001) |  | 0.1562 (<.0001) |
|  | NLB | 0.8357 (<.0001) | -0.8383 (<.0001) |  | 0.8112 (<.0001) |
|  | Mediator (*M*):Maternal age (Event/Ref: ≥ 20) |  |  |  |  |
|  | <12 years |  |  | 0.3472 (<.0001) | 0.2417 (<.0001) |
| 2 | Independent (*X*): Race- Nativity (Ref: NLW) |  |  |  |  |
|  | BLFB | 0.1974 (<.0001) | -1.5657 (<.0001) |  | 0.1122 (0.0003) |
|  | BLUSB | 0.5937 (<.0001) | -1.0448 (<.0001) |  | 0.5468 (<.0001) |
|  | WLFB | 0.00392 (0.4663) | -2.1940 (<.0001) |  | -0.1327 (<.0001) |
|  | WLUSB | 0.1935 (<.0001) | -1.0568 (<.0001) |  | 0.1445 (<.0001) |
|  | NLB | 0.8357 (<.0001) | -0.8855 (<.0001) |  | 0.7979 (<.0001) |
|  | Mediator (*M*): Maternal education (Event/Ref: ≥ 12) |  |  |  |  |
|  | <12 |  |  | 0.3478 (<.0001) | 0.3332 (<.0001) |
| 3 | Independent (*X*): Race- Ethnicity-Nativity (Ref: NLW) |  |  |  |  |
|  | BLFB | 0.1974 (<.0001) | -1.2966 (<.0001) |  | 0.0666 (0.0298) |
|  | BLUSB | 0.5937 (<.0001) | -1.9679 (<.0001) |  | 0.4073 (<.0001) |
|  | WLFB | 0.00392 (0.4663) | -0.7529 (<.0001) |  | -0.0723 (<.0001) |
|  | WLUSB | 0.1935 (<.0001) | -1.1309 (<.0001) |  | 0.0789 (<.0001) |
|  | NLB | 0.8357 (<.0001) | -1.8057 (<.0001) |  | 0.6621 (<.0001) |
|  | Mediator (*M*): Marital status (Event/Ref: Yes) |  |  |  |  |
|  | No |  |  | 0.5762 (<.0001) | 0.4192 (<.0001) |
| 4 | Independent (*X*): Race- Ethnicity- Nativity (Ref: NLW) |  |  |  |  |
|  | BLFB | 0.1974 (<.0001) | 1.2301 (<.0001) |  | 0.0745 (0.0153) |
|  | BLUSB | 0.5937 (<.0001) | 1.6139 (<.0001) |  | 0.3995 (<.0001) |
|  | WLFB | 0.00392 (0.4663) | 0.8131 (<.0001) |  | -0.0608 (<.0001) |
|  | WLUSB | 0.1935 (<.0001) | 1.1206 (<.0001) |  | 0.0848 (<.0001) |
|  | NLB | 0.8357 (<.0001) | 1.3373 (<.0001) |  | 0.6348 (<.0001) |
|  | Mediator (*M*): Paternal acknowledgement (Event/Ref: Married) |  |  |  |  |
|  | No paternal acknowledgement |  |  | 0.8006 (<.0001) | 0.5680 (<.0001) |
|  | Unmarried but paternal acknowledgement |  |  | 0.4603 (<.0001) | 0.3492 (<.0001) |
| 5 | Independent (*X*): Race- Ethnicity- Nativity (Ref: NLW) |  |  |  |  |
|  | BLFB | 0.1974 (<.0001) | 1.7674 (<.0001) |  | 0.1331 (<.0001) |
|  | BLUSB | 0.5937 (<.0001) | 1.5213 (<.0001) |  | 0.5351 (<.0001) |
|  | WLFB | 0.00392 (0.4663) | 1.8614 (<.0001) |  | -0.0671 (<.0001) |
|  | WLUSB | 0.1935 (<.0001) | 1.3681 (<.0001) |  | 0.1384 (<.0001) |
|  | NLB | 0.8357 (<.0001) | 1.4703 (<.0001) |  | 0.7771 (<.0001) |
|  | Mediator (*M*): WIC status (Event/Ref: Yes) |  |  |  |  |
|  | No |  |  | -0.2930 (<.0001) | -0.1630 (<.0001) |

BLFB, Black Latina foreign-born; BLUSB, Black Latina US-born; WLFB, White Latina foreign-born; WLUSB, White Latina US-born; NLB, Non-Latina Black; NLW, Non-Latina White.

^a^Study population: 7,865,264 singleton births of Black and White women in 36 states and District of Columbia with non-imputed maternal race, with data for nativity and all selected risk factors in the study (Main manuscript Figure 1).

^*^b is the estimated value of the regression coefficient of interdependent variable (*X*) or mediator variable (*M*).

**Online Resource Table 5.** Distribution of Infant Low Birth Weight Rates Among Latina and Non-Latina Mothers by Race and Nativity in the Study Population,^a^ United States, 2011-2013

| **Study Groups by Race, Ethnicity,**  **and Nativity** | **LBW Infant (<2500g)** | |
| --- | --- | --- |
|  | Yes  *n* (%) | No  *n* (%) |
| Black Latina US-Born | 2,980 (8.9) | 30,689 (91.2) |
| Black Latina Foreign-Born | 1,144 (6.1) | 17,512 (93.9) |
| White Latina US-Born | 53,751 (6.1) | 826,016 (93.9) |
| White Latina Foreign-Born | 42,687 (5.1) | 792,894 (94.9) |
| Non-Latina White US-Born | 236,145 (5.2) | 4,353,193 (94.9) |
| Non-Latina White Foreign-Born | 12,334 (4.2) | 280,325 (95.8) |
| Non-Latina Black US-Born | 118,858 (11.6) | 903,473 (88.4) |
| Non-Latina Black Foreign-Born | 14,288 (7.6) | 173,146 (92.4) |

Abbreviation: LBW, low birth weight.

^a^Study population: 7,865,264 singleton births of Black and White women in 36 states and District of Columbia with non-imputed maternal race, with data for nativity and all selected risk factors in the study (Main manuscript Figure 1).

**Online Resource Table 6.** Distribution of Infant Low Birth Weight Rates Among Latina and Non-Latina Mothers by Nativity in the Study Population,^a^ United States, 2011-2013

| **Study Groups by Ethnicity and Nativity** | **LBW Infant (<2500g)** | |
| --- | --- | --- |
|  | Yes  *n* (%) | No  *n* (%) |
| Latina US-Born | 56,731 (6.2) | 856,705 (93.8) |
| Latina Foreign-Born | 43,831 (5.1) | 810,406 (94.9) |
| Non-Latina US-Born | 355,003 (6.3) | 5,256,666 (93.7) |
| Non-Latina Foreign-Born | 26,622 (5.6) | 453,471 (94.5) |

Abbreviation: LBW, low birth weight.

^a^Study population: 7,865,264 singleton births of Black and White women in 36 states and District of Columbia with non-imputed maternal race, with data for nativity and all selected risk factors in the study (Main manuscript Figure 1).

**Online Resource Table 7.** Distribution of Infant Low Birth Weight Rates Among Latina and Non-Latina Mothers by Race in the Study Population,^a^ United States, 2011-2013

| **Study Groups by Ethnicity and Race** | **LBW Infant (<2500g)** | |
| --- | --- | --- |
|  | Yes  *n* (%) | No  *n* (%) |
| Black Latina | 4,124 (7.9) | 48,201 (92.1) |
| White Latina | 96,438 (5.6) | 1,618,910 (94.4) |
| Non-Latina White | 248,479 (5.1) | 4,633,518 (94.9) |
| Non-Latina Black | 133,146 (11.0) | 1,076,619 (89.0) |

Abbreviation: LBW, low birth weight.

^a^Study population: 7,865,264 singleton births of Black and White women in 36 states and District of Columbia with non-imputed maternal race, with data for nativity and all selected risk factors in the study (Main manuscript Figure 1).

**Online Resource Table 8**. Distribution (%) of Selected Risk Factors Among Black Latina US-Born, Black Latina Foreign-Born, White Latina US-Born, White Latina Foreign-Born, Non-Latina White and Non-Latina Black Mothers Using Combined Race Data^a^, United States, 2011-2013

|  | **Black Latina** | |  | **White Latina** | | **Non-Latina**  **White**  ***n*= 4,901,612**  **%** | **Non-Latina**  **Black**  ***n*=1,216,219**  **%** | **Effect size^d^**  **(LD^e^)** |
| --- | --- | --- | --- | --- | --- | --- | --- | --- |
| **Risk Factor** | **US Born**  ***n*=56,608**  **%** | **Foreign Born**  ***n*=50,984**  **%** |  | **US Born**  ***n*=1,037,754**  **%** | **Foreign Born**  ***n*=1,130,419**  **%** |  |  |  |
| Maternal age < 20 years | 25^b,c^ | 11 |  | 24 | 10 | 9 | 19 | 0.17 (2)^*^ |
| High parity | 12 | 15 |  | 12 | 19 | 10 | 15 | 0.09 (3)^*^ |
| Maternal education <12 years | 23 | 44 |  | 23 | 49 | 9 | 20 | 0.35 (2)^**^ |
| Unmarried | 73 | 60 |  | 57 | 48 | 30 | 72 | 0.33 (2)^**^ |
| Unmarried and no paternal acknowledgement | 19 | 13 |  | 14 | 10 | 8 | 32 | 0.25 (3)^**^ |
| No WIC recipient | 31 | 26 |  | 35 | 25 | 68 | 33 | 0.37 (2)^**^ |
| Medical disease during pregnancy | 10 | 12 |  | 9 | 10 | 11 | 13 | 0.04 (2)^$^ |
| No first trimester initiation of prenatal care | 35 | 37 |  | 29 | 33 | 21 | 37 | 0.14 (2)^*^ |
| Smoke Cigarettes | 7 | 1 |  | 3 | 0.5 | 13 | 8 | 0.17 (2)^*^ |
| Normal BMI (18.5−24.9) | 39 | 43 |  | 41 | 44 | 50 | 37 | 0.07 (3)^*^ |

$Negligible effect size, * small effect size, ** medium effect size.

Abbreviations: WIC, Special Supplemental Nutrition for Women, Infants and Children.

^a^Combined race data consists of data from both imputed and non-imputed race populations that excludes observations for non-singleton, missing race/nativity, Asian/Pacific Islander and Indian/Alaskan Native, and 14 states that did not collect data for 1 or more variable(s) used in the study or collected data in a nonstandard format.

^b^The *P-*value of chi-square test of association among 6 groups of women is <0.0001 for all risk factors.

^c^Percentages for all risk factors rounded to the nearest integer.

^d^Cramer’s V statistic is used as a measure of effect size.

^e^LD is the smaller/lower dimension of a (r×c) cross table.

**
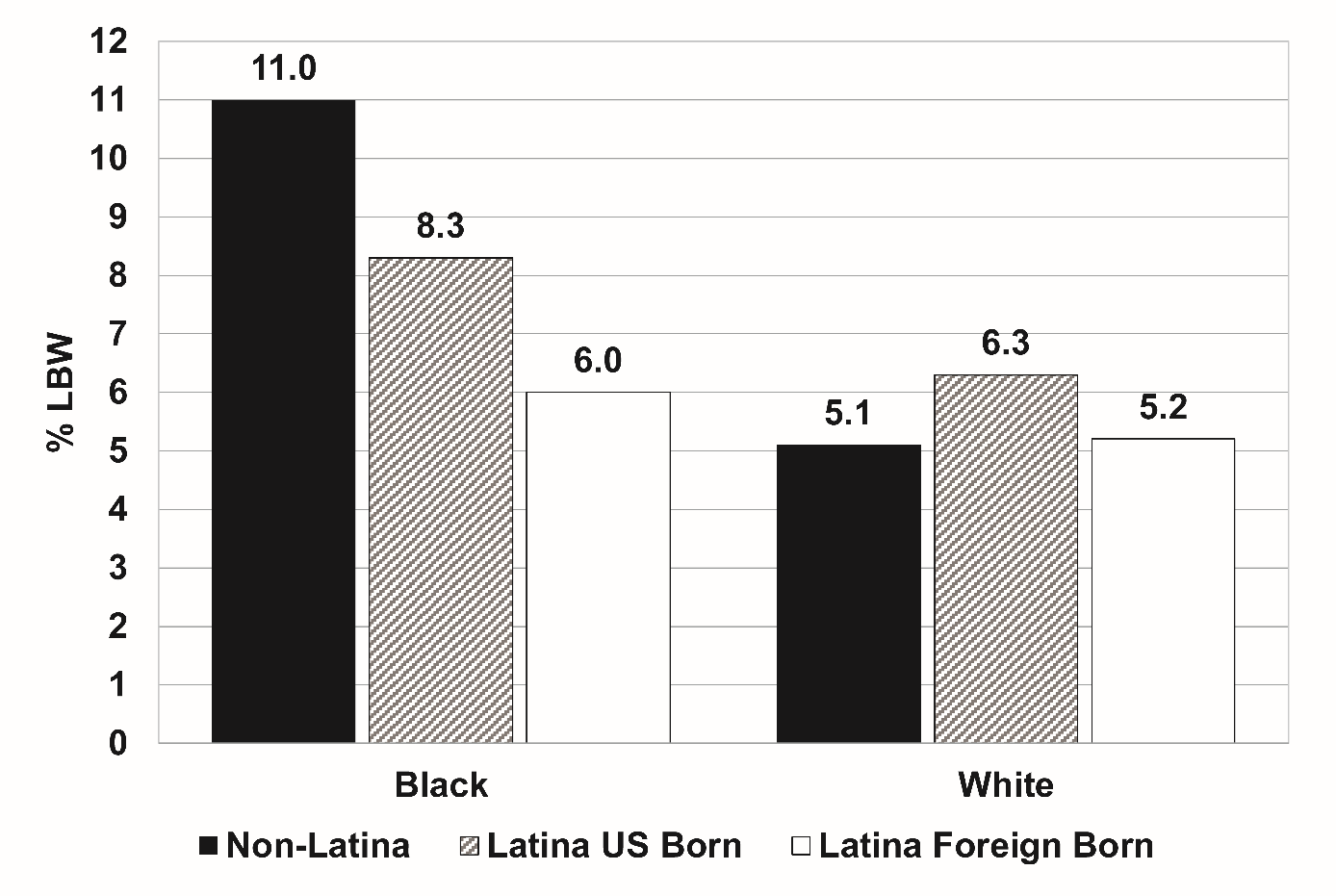
**

**Online Resource Figure 1.**  Distribution of infant low birth weight rates among Black and White mothers according to nativity and Latina/non-Latina ethnicity, combined race data, United States, 2011-2013. Combined race data consists of data from both imputed and non-imputed race populations that excludes observations for non-singleton, missing race/nativity, Asian/Pacific Islander and Indian/Alaskan Native, and 14 states that did not collect data for 1 or more variable(s) used in the study or collected data in a nonstandard format.

**Online Resource Table 9.** Odds Ratios for the Effect of Maternal Ethnicity, Nativity, and Race on Infant Low Birth Weight Among Latina and Non-Latina Black and White Mothers, Adjusting for Different Sets of Risk Factors in Multivariable Logistic Regression Models Using Combined Race Data^a^ , United States, 2011-2013

| **Study groups by**  **Race, Ethnicity, and**  **Nativity** | **Model 1^b^**  ***n* = 8,387,211** | |  | **Model 2^c^ *n* = 8,187,789** | |  | **Model 3^d^ *n* = 7,721,768** | |  | **Model 4^e^ *n* = 7,721,768** | |
| --- | --- | --- | --- | --- | --- | --- | --- | --- | --- | --- | --- |
|  | **OR** | **95% CI** |  | **OR** | **95% CI** |  | **OR** | **95% CI** |  | **OR** | **95% CI** |
| **Non-Latina White** | **1.00** |  |  | **1.00** |  |  | **1.00** |  |  | **1.00** |  |
| Non-Latina Black | 2.30*** | 2.29, 2.32 |  | 1.89*** | 1.88,1.91 |  | 1.94*** | 1.93, 1.96 |  | 2.22*** | 2.20, 2.23 |
| Black Latina US-Born | 1.69*** | 1.64, 1.74 |  | 1.41*** | 1.37, 1.46 |  | 1.49*** | 1.45, 1.55 |  | 1.72*** | 1.66, 1.77 |
| Black Latina Foreign-Born | 1.19*** | 1.14, 1.23 |  | 1.00 | 0.97,1.04 |  | 1.04 | 0.99, 1.08 |  | 1.27*** | 1.22, 1.32 |
| White Latina US-Born | 1.24*** | 1.23, 1.25 |  | 1.09*** | 1.08, 1.10 |  | 1.18*** | 1.17, 1.19 |  | 1.37*** | 1.35, 1.38 |
| White Latina Foreign-Born | 1.03*** | 1.02, 1.04 |  | 0.89*** | 0.88, 0.90 |  | 0.94*** | 0.93, 0.95 |  | 1.15*** | 1.13, 1.16 |

* *P* < 0.05, ** *P* < 0.01, *** *P* < 0.001

Abbreviations: CI, confidence interval; OR, odds ratio; WIC, Special Supplemental Nutrition for Women, Infants
and Children

^a^Combined race data consists of data from both imputed and non-imputed race populations that excludes observations for non-singleton, missing race/nativity, Asian/Pacific Islander and Indian/Alaskan Native, and 14 states that did not collect data for 1 or more variable(s) used in the study or collected data in a nonstandard format.

^b^ Model 1, a crude analysis (unadjusted model), included only the Group variable (the Group variable represents the 6 study groups of race, ethnicity, and nativity categories).

^c^ Model 2 adjusted for maternal age, maternal education, marital status, paternal acknowledgment, and WIC receipt.

^d^Model 3 adjusted for Model 2 covariates plus sex of infant, first trimester initiation, parity, mother’s medical disease during pregnancy, and BMI.

^e^ Model 4 adjusted for Model 3 covariates plus cigarette smoking.

**Online Resource Table 10**. Distribution (%) of Selected Risk Factors Between Non-Imputed and Imputed for Black Latina US-Born and Black Latina Foreign-Born using Combined Race Data^a^, United States, 2011-2013

|  | **Black Latina US Born** | | | |  | **Black Latina Foreign** | | | |
| --- | --- | --- | --- | --- | --- | --- | --- | --- | --- |
| **Risk Factor** | **Non-Imputed**  ***n* (%)** | **Imputed**  ***n* (%)** | **Chi-square**  **(P-value)** | **Effect size^b^**  **(LD^c^)** |  | **Non-Imputed**  ***n* (%)** | **Imputed**  ***n* (%)** | **Chi-square**  **(P-value)** | **Effect size^b^**  **(LD^c^)** |
| Maternal age |  |  |  |  |  |  |  |  |  |
| < 20 years | 8725 (25.90) | 5631 (24.57) | 12.7018  (0.0004) | 0.0150 (2)^$^ |  | 1773 (9.50) | 3766 (11.65) | 56.9459  (<.0001) | 0.0334 (2)^$^ |
| >= 20 years | 24965 (74.10) | 17287 (75.43) |  |  |  | 16898 (90.50) | 28547 (88.35) |  |  |
| Parity |  |  |  |  |  |  |  |  |  |
| Primiparous | 15423 (45.99) | 9905 (43.44) | 35.7224  (<.0001) | 0.0252 (2)^$^ |  | 6806 (36.58) | 9195 (28.66) | 590.8204  (<.0001) | 0.1080 (2)^*^ |
| Low parity | 14249 (42.49) | 10154 (44.53) |  |  |  | 9803 (52.69) | 17293 (53.90) |  |  |
| High parity | 3866 (11.53) | 2745 (12.04) |  |  |  | 1997 (10.73) | 5595 (17.44) |  |  |
| Maternal education |  |  |  |  |  |  |  |  |  |
| <12 years | 7456 (22.25) | 5676 (24.87) | 52.1545  (<.0001) | 0.0304 (2)^$^ |  | 6047 (32.51) | 16371 (51.21) | 1665.3631  (<.0001) | 0.1815 (2)^*^ |
| >=12 years | 26054 (77.75) | 17146 (75.13) |  |  |  | 12551 (67.49) | 15597 (48.79) |  |  |
| Marital status |  |  |  |  |  |  |  |  |  |
| Married | 8419 (24.99) | 6843 (29.86) | 164.2074  (<.0001) | 0.0539 (2)^$^ |  | 7368 (39.46) | 12896 (39.91) | 0.9889  (0.3200) | 0.0044 (2)^$^ |
| Unmarried | 25271 (75.01) | 16075 (70.14) |  |  |  | 11303 (60.54) | 19417 (60.09) |  |  |
| Paternal acknowledgement |  |  |  |  |  |  |  |  |  |
| Married | 8419 (25.18) | 6843 (30.02) | 2042.3640  (<.0001) | 0.1906 (2)^*^ |  | 7368 (39.84) | 12896 (40.08) | 181.7888  (<.0001) | 0.0599 (2)^$^ |
| Unmarried but paternal acknowledgement | 16719 (50.01) | 13745 (60.30) |  |  |  | 8183 (44.25) | 15470 (48.07) |  |  |
| Unmarried and no paternal acknowledgement | 8292 (24.80) | 2206 (9.68) |  |  |  | 2941 (15.90) | 3813 (11.85) |  |  |
| WIC recipient |  |  |  |  |  |  |  |  |  |
| Yes | 22646 (68.23) | 15955 (70.42) | 30.3525  (<.0001) | 0.0233 (2)^$^ |  | 13413 (73.31) | 23766 (75.04) | 18.2082  (<.0001) | 0.0191 (2)^$^ |
| No | 10545 (31.77) | 6701 (29.58) |  |  |  | 4883 (26.69) | 7905 (24.96) |  |  |
| Gender of infant |  |  |  |  |  |  |  |  |  |
| Female | 16463 (48.87) | 11226 (48.98) | 0.0750  (0.7842) | 0.0012 (2)^$^ |  | 9199 (49.27) | 15775 (48.82) | 0.9570  (0.3279) | 0.0043 (2)^$^ |
| Male | 17227 (51.13) | 11692 (51.02) |  |  |  | 9472 (50.73) | 16538 (51.18) |  |  |
| Medical disease during pregnancy |  |  |  |  |  |  |  |  |  |
| Yes | 313 (9.36) | 2352 (10.28) | 12.9122  (0.0003) | 0.0151 (2)^$^ |  | 2042 (11.08) | 3783 (11.75) | 5.2135  (0.0224) | 0.0101 (2)^$^ |
| No | 30390 (90.64) | 20538 (89.72) |  |  |  | 16388 (88.92) | 28405 (88.25) |  |  |
| First trimester initiation of prenatal care |  |  |  |  |  |  |  |  |  |
| Yes | 21386 (65.77) | 14363 (64.83) | 5.1443  (0.0233) | 0.0097 (2)^$^ |  | 12376 (68.80) | 18142 (58.85) | 480.3366  (<.0001) | 0.10 (2)^*^ |
| No | 11132 (34.23) | 7793 (35.17) |  |  |  | 5612 (31.20) | 12687 (41.15) |  |  |
| Smoke Cigarettes |  |  |  |  |  |  |  |  |  |
| Yes | 2644 (7.93) | 1315 (5.78) | 95.1296  (<.0001) | 0.0412 (2)^$^ |  | 151 (0.82) | 370 (1.15) | 12.6733  (0.0004) | 0.0158 (2)^$^ |
| No | 30704 (92.07) | 21434 (94.22) |  |  |  | 18240 (99.18) | 31694 (98.85) |  |  |
| Body mass index (BMI) |  |  |  |  |  |  |  |  |  |
| Underweight (BMI <18.5) | 1310 (4.02) | 699 (3.15) | 82.0980  (<.0001) | 0.0387 (2)^$^ |  | 597 (3.33) | 880 (2.96) | 103.9289  (<.0001) | 0.0467 (2)^$^ |
| Normal (BMI 18.5-24.9) | 13104 (40.25) | 8361 (37.62) |  |  |  | 8258 (46.07) | 12398 (41.74) |  |  |
| Overweight (BMI 25.0-29.0) | 8717 (26.77) | 6211 (27.95) |  |  |  | 5435 (30.32) | 9681 (32.59) |  |  |
| Obesity I (BMI 30.0-34.9) | 5067 (15.56) | 3759 (16.91) |  |  |  | 2410 (13.44) | 4518 (15.21) |  |  |
| Obesity II (BMI 35.0-39.9) | 2527 (7.76) | 1833 (8.25) |  |  |  | 820 (4.57) | 1438 (4.84) |  |  |
| Extreme Obesity III (BMI >=40.0) | 1835 (5.64) | 1362 (6.13) |  |  |  | 405 (2.26) | 788 (2.65) |  |  |

$Negligible effect size, * small effect size.

Abbreviations: LD, lower dimension; WIC, Special Supplemental Nutrition for Women, Infants and Children.

^a^Combined race data consists of data from both imputed and non-imputed race populations that excludes observations for non-singleton, missing race/nativity, Asian/Pacific Islander and Indian/Alaskan Native, and 14 states that did not collect data for 1 or more variable(s) used in the study or collected data in a nonstandard format.

^b^ Cramer’s V statistic is used as a measure of effect size.

^c^LD is the lower dimension of a (r×c) cross table.

**Online Resource Table 11.** Distribution of Total Singleton Births According to Mother’s Nativity, Ethnicity and Race in Restricted and Unrestricted NCHS Natality Data With Imputed and Non-Imputed Race United States, 2011-2013

| **Mother's Nativity** | **Ethnicity** | **Race** | **Unrestricted Data^a^**  ***n* (%)** | **Restricted**  **Data^b^**  ***n* (%)** |
| --- | --- | --- | --- | --- |
| US Born | Latina | Black | 68,069 (0.6) | 56,608 (0.6) |
|  |  | White | 1,167,970 (10.3) | 1,037,754 (11.4) |
|  |  | Asian/Pacific Islander | 16,008 (0.1) | 10,158 (0.1) |
|  |  | Indian/Alaskan Native | 13,433 (0.1) | 11,931 (0.1) |
|  |  | **Subtotal** | **1,265,480 (11.2)** | **1,116,451** (**12.3**) |
|  | Non-Latina | Black | 1,436,156 (12.7) | 1,026,298 (11.3) |
|  |  | White | 5,772,649 (50.9) | 4,601,231 (50.6) |
|  |  | Asian/Pacific Islander | 147,927 (1.3) | 112,799 (1.2) |
|  |  | Indian/Alaskan Native | 111,691 (1.0) | 81,669 (0.9) |
|  |  | **Subtotal** | **7,468,423 (65.9)** | **5,821,997** (**64.0**) |
|  | **Total US Born** |  | **8,733,903 (77.1)** | **6,938,448** (**76.3**) |
| Foreign Born | Latina | Black | 64,421 (0.6) | 50,984 (0.6) |
|  |  | White | 1,325,648 (11.7) | 1,130,419 (12.4) |
|  |  | Asian/Pacific Islander | 13,682 (0.1) | 11,015 (0.1) |
|  |  | Indian/Alaskan Native | 6,485 (0.1) | 5,782 (0.1) |
|  |  | **Subtotal** | **1,410,236 (12.5)** | **1,198,200** (**13.2**) |
|  | Non-Latina | Black | 233,800 (2.1) | 189,921 (2.1) |
|  |  | White | 379,367 (3.4) | 300,381 (3.3) |
|  |  | Asian/Pacific Islander | 574,927 (5.1) | 470,076 (5.2) |
|  |  | Indian/Alaskan Native | 1,947 (0.0) | 1,122 (0.0) |
|  |  | **Subtotal** | **1,190,041** **(10.5**) | **961,500** (**10.6**) |
|  | **Total Foreign Born** |  | **2,600,277** **(23.0)** | **2,159,700** (**23.7**) |
| **Grand total** |  |  | **11,334,180** **(100)** | **9,098,148** (**100**) |

^a^Unrestricted data contains 2011-2013 NCHS natality data for singleton live births of US-born and foreign-born mothers (excluding unknown/missing nativity) for all 50 states (including the 14 states with inadequate data) plus the District of Columbia.

^b^Restricted data contains 2011-2013 NCHS natality data for singleton live births of US-born and foreign-born mothers (excluding unknown/missing nativity) for 36 states plus District of Columbia; the 14 states excluded from the study either did not collect data for one or more study variable(s) or collected data in a nonstandard format (MI [Osterman et al. 2013] and GA, for smoking).

**Online Resource Table 12.** Summary of Analyzed and Missing Observations of Selected Variables in Combined Race Data^a^, United States, 2011-2013

| **Variable** | **Observations** | |
| --- | --- | --- |
|  | **Analyzed**^b^  ***n* (%)** | **Missing**  ***n* (%)** |
| Low Birth Weight | 8,387,211 (99.9) | 6,385 (0.1) |
| Race and nativity | 8,393,596 (100.0) | 0 (0.0) |
| Maternal Age | 8,393,596 (100.0) | 0 (0.0) |
| Maternal Education | 8,338,879 (99.4) | 54,717 (0.7) |
| Marital Status | 8,393,596 (100.0) | 0 (0.0) |
| Parity | 8,354,231 (99.5) | 39,365 (0.5) |
| First Trimester Initiation | 8,146,686 (97.1) | 246,910 (2.9) |
| Paternal Acknowledgement | 8,369,542 (99.7) | 24,054 (0.3) |
| WIC Recipient | 8,257,328 (98.4) | 136,268 (1.6) |
| Gender of Infant | 8,393,596 (100.0) | 0 (0.0) |
| Medical Disease During Pregnancy | 8,358,900 (99.6) | 34,696 (0.4) |
| Body Mass Index | 8,111,736 (96.6) | 281,860 (3.4) |
| Smoking cigarettes | 8,335,306 (99.3) | 58,290 (0.7) |

Abbreviation: WIC, Special Supplemental Nutrition for Women, Infants and Children

^a^Combined race data consists of data from both imputed and non-imputed race populations that excludes observations for non-singleton, missing race/nativity, Asian/Pacific Islander and Indian/Alaskan Native, and 14 states that did not collect data for 1 or more variable(s) used in the study or collected data in a nonstandard format.

^b^No missing observations.

**Online Resource Appendix**

**Complexity of self-identification of race among Latino populations**

Both the US Census reports and NCHS natality data reflect the complexity and ambiguity of race classification of Latino populations. In the federal statistical system, Latino origin is different from race (Humes et al. 2011; Rios et al. 2014; CDC 2013). When completing federal census forms, respondents are instructed to indicate their ethnicity (typically Latino or non-Latino) and race separately; race options include the 5 major OMB (Office of Management and Budget) categories (White, Black or African American, American Indian and Alaska Native, Asian, and Native Hawaiian and Other Pacific Islander). Respondents who do not identify themselves with any of the 5 OMB race classifications have an option to identify as “Some Other Race.” When the data is compiled, subjects with missing data on the race component are placed in the “Some Other Race” group. This grouping of missing data as Some Other Race introduces ambiguity in race reporting of Latina populations. For example, *Race Reporting Among Hispanics: 2010* reports that the Some Other Race category corresponds to 36.7% of the Latino population (Rios et al. 2014) and 63.3% clearly self-identify as 1 or more of the 5 listed races. Among the clearly self-identified Latino population, 53.0% identify as White and 2.5% identify as Black. In contrast, 96.1% of non-Latinos specified only 1 race (Humes et al. 2011; Rios et al. 2014).

**Race identification in natality data**

The representation of race among Latina populations in NCHS natality data is based on more assumptions than the census reports. The most recent census forms highlight the distinction between ethnicity and race, stating clearly that Hispanic origin is not a race, with explicit instructions to answer both the question about Hispanic origin and the question about race (Humes et al. 2011). The CDC/NCHS relies on data from birth certificates to determine maternal race; although Hispanic origin and race are 2 separate questions, the forms do not have the explicit reminder to answer both questions (CDC 2013). Also, NCHS natality data only reports 4 race categories (White, Black or African American, American Indian and Alaska Native, and Asian/ Pacific Islander). When tabulating maternal race data from the birth certificates, women who identify as “Some Other Race,” or have missing data on race are assigned to 1 of the 4 OMB races using statistical methods such as imputation (CDC 2013; Rubin and Schenker 1991). When we analyzed our NCHS natality data, our race classification numbers were similar to the census reports. We found that 77.7% of Latina mothers have a race based on self-identification (non-imputed race), while 22.3% of Latinas were assigned a race using imputation (imputed race). When split by White and Black race, 79.5 % of White Latinas had non-imputed race, compared to 51% of Black Latinas. In non-Latina populations, the vast majority (over 99%) of Black and White mothers had non-imputed race (Online Resource Table 2).

**Bridged Race**

Women who identify as more than 1 race on the birth certificate are assigned a single race through a process called bridging (CDC 2013).). In our study we did not exclude mothers with bridged race because these mothers did identify themselves with a race; moreover, of our study population, only 1.91% had bridged race. We are confident that the inclusion of the small number of mothers with bridged race did not affect our findings, given that the results of our combined analysis (reincorporating mothers with imputed race; Online Resource Tables 7 to 10 and Online Resource Figure) are very similar to our analysis of the study population (restricted to mothers with non-imputed race).
